# Factors associated with in-hospital recurrence of intestinal intussusception in children

**DOI:** 10.1186/s12887-023-04267-9

**Published:** 2023-08-26

**Authors:** Jing Zhang, Qi Dong, Xiaoxia Su, Junshan Long

**Affiliations:** Department of General Surgery, Hainan Women and Children’s Medical Center, Haikou, 570100 China

**Keywords:** Intussusception, Intestinal, Infant, Child, Risk factors, Recurrence

## Abstract

**Background:**

A minority of children experience in-hospital recurrence of intestinal intussusception after treatment. This study investigated the factors associated with in-hospital recurrence of intussusception in pediatric patients in China.

**Methods:**

This retrospective study included patients aged 0–18 years-old with intestinal intussusception treated at Hainan Women and Children’s Medical Center between January 2019 and December 2019. Demographic and clinical characteristics were extracted from the medical records. Factors associated with in-hospital recurrence of intussusception were identified by logistic regression analysis.

**Results:**

The analysis included 624 children (400 boys) with a median age of 1.8 years (range, 2 months and 6 days to 9 years). Seventy-three children (11.7%) had in-hospital recurrence of intussusception after successful reduction with air enema. Multivariate logistic regression analysis identified age > 1 year-old (odds ratio [OR]: 7.65; 95% confidence interval [95%CI]: 2.70–21.71; *P* < 0.001), secondary intestinal intussusception (OR: 14.40; 95%CI: 4.31–48.14; *P* < 0.001) and mesenteric lymph node enlargement (OR: 1.90; 95%CI: 1.13–3.18; *P* = 0.015) as factors independently associated with in-hospital recurrence of intussusception.

**Conclusions:**

Age > 1 year-old, secondary intussusception and mesenteric lymph node enlargement were independently associated with increased odds of in-hospital recurrence of intussusception after successful reduction with air enema.

## Background

Intussusception describes the intussusception of a segment of intestine into the lumen of a more distant segment of the bowel and is one of the most common acute abdominal disorders in infancy [1]. Intestinal intussusception occurs most frequently at the age of 6 months [1], and the incidence of intussusception in China during 2009–2013 was estimated to be 112.9 per 100,000 children aged < 2 years-old [2]. Intussusception in children is a medical emergency that necessitates timely diagnosis and treatment [3]. The main presenting symptoms of intestinal intussusception in children under 2 years of age include vomiting, abdominal pain and blood in the stools [4]. The diagnosis of intussusception is most often made using ultrasonography, but other useful investigations include X-ray, computed tomography and barium/air enema [1, 5, 6]. Management strategies include reduction by pneumatic (air) enema, hydrostatic (liquid) enema, laparoscopic surgery and open surgery [3, 7, 8].

Previous research has indicated that a recurrence of intussusception occurs in 8–19% of children [9–13]. Several clinical analyses have attempted to identify factors associated with an increased risk of intussusception recurrence. Many investigations have concluded that older age (≥ 1 year-old or ≥ 2 years-old, depending on the analysis) is associated with elevated odds of intussusception recurrence [13–16]. Pathological lead points (PLPs), such as Meckel’s diverticulum, duplication, polyps or tumors, have also been reported as independent predictors of recurrent intussusception [13, 14, 16]. Other factors reported to increase the risk of intussusception recurrence include an absence of vomiting [13], rectal bleeding [16], fever [17], surgical treatment [18], overweight/obesity and white blood cell (WBC) count ≥ 20 × 10^9^ /L [19]. However, there have also been disagreements between studies. For example, Guo et al. found that symptom duration ≤ 12 h and mass located in the right abdomen were independent predictors of recurrent intussusception [13], whereas the factors identified by Xie et al. included duration of symptoms ≥ 48 h and mass located in the left abdomen [16]. Furthermore, Zhang et al. demonstrated that mass diameter > 2.55 cm and enlarged abdominal lymph nodes were related to recurrence of intussusception [20], whereas Vo et al. did not identify any radiologic features that were associated with early recurrence, although the risk of recurrence was higher in females and patients with fever [17]. A recent meta-analysis by Ye et al. identified only fever, absence of vomiting and PLPs as factors associated with a higher risk of recurrence of intestinal intussusception [21].

At least half of all recurrences of intussusception occur within 48 h after treatment [14, 17, 22, 23], with a median time to recurrence of 25 h after fluoroscopy-guided air enema [15]. Furthermore, Lee et al. reported that in-hospital recurrence of intussusception occurred in 6.1% of pediatric patients [24]. Knowledge of the risk factors for early recurrence of intestinal intussusception would help pediatric surgeons to identify those patients requiring careful observation during the early post-operative period. However, only a relatively small number of studies have evaluated the clinical factors associated with an increased risk of intussusception recurrence, but the findings have varied. Simanovsky et al. found that patients with early recurrence were older than those who did not have early recurrence (23 months-old vs. 12 months-old) [25], while Shen et al. concluded that age ≥ 2 years-old and absence of fever were associated with short-term recurrence [26]. Another analysis highlighted higher body weight, PLPs and onset during the second quarter of the year as factors predictive of early recurrence [27]. Additionally, a recent study by Zhu et al. showed that a blood level of monocyte chemotactic protein-1 exceeding 23.24 ng/mL predicted short-term recurrence of intussusception with a sensitivity of 82.1% and a specificity of 75.7% [28]. However, Cho et al. did not find any differences in age, reduction success rate, treatment method (surgery vs. non-surgery) or PLPs between patients with recurrence within 48 h and those without recurrence [29]. Thus, the findings of these studies are not entirely consistent.

The aim of this study was to investigate the factors associated with in-hospital recurrence of intestinal intussusception in pediatric patients in China. To achieve this aim, we performed a retrospective analysis of data for patients aged ≤ 18 years-old who were treated for intussusception at our institution over a 12-month period.

## Methods

### Study design and patients

This retrospective study included patients with intestinal intussusception treated at Hainan Women and Children’s Medical Center (Haikou, Hainan, China) between January 2019 and December 2019. The inclusion criteria were as follows: (1) aged 0–18 years-old; (2) the diagnosis of intussusception was confirmed by air enema imaging; and (3) intussusception was successfully reduced by air enema. Intussusception was defined as intestinal obstruction caused by the insertion of a segment of intestine and its corresponding mesentery into the adjacent intestinal cavity. There were no formal exclusion criteria. The study protocol was approved by the Ethics Committee of Hainan Women and Children’s Medical Center (HNWCMC Tribunal No. [26], 2023). The requirement for informed consent was waived because the analysis was retrospective and anonymized.

### Data collection and definitions

The following demographic, clinical and ultrasonographic characteristics were obtained from the medical record system: age, sex, symptoms (including fever, vomiting and bloody stools), duration of symptoms prior to admission, history of previous intussusception, type of intestinal intussusception (primary [idiopathic] or secondary to an identifiable organic lesion), site of the lesion (ileocecal junction, ascending colon, hepatic flexure, transverse colon, splenic flexure or other), lesion size (inner diameter and outer diameter), mesenteric lymph node (enlargement or normal), C-reactive protein (CRP) level, WBC count, neutrophil percentage and lymphocyte percentage. The normal mesenteric lymph node size of the general pediatric population was 0.2-0.5 cm. There were more than 1 lymph node in the mesentery in the same area, with a short axis diameter > 0.5 cm, long axis diameter > 1.0 cm or aspect ratio > 2, or lymph nodes arranged in clusters, Color Doppler flow imaging showing increased blood flow in lymph nodes, were considered mesenteric lymph node enlargement. Recurrence of intussusception specifically referred to the recurrence of intussusception during hospitalization.

### Statistical analysis

SPSS 26.0 software was used for the analysis. Normally distributed continuous data are described as the mean ± standard deviation (SD) and were compared between groups using Student’s t-test for independent samples. Non-normally distributed continuous data are presented as median (interquartile range [IQR]) and were analyzed using the Mann-Whitney U test. Categorical data are described as *n* (%) and were compared between groups using the chi-squared test or Fisher’s exact test. Logistic regression analysis was used to identify factors associated with in-hospital recurrence of intussusception, and variables with statistical significance in the univariate analysis were included in the multivariate analysis. Odds ratios (ORs) and 95% confidence intervals (95%CIs) were calculated. A two-sided *P*-value < 0.05 was considered statistically significant.

## Results

### Baseline characteristics of the study participants

The analysis included 624 children (400 boys, 64.1%) with a median age of 1.8 years (range, 2 months and 6 days to 9 years). The age at presentation was ≤ 1 year-old in 178 children (28.5%), > 1 year-old in 446 children (71.5%), among them, ≥ 2 years-old in 255 children (40.9%). Seventy-four children (11.9%) had a history of previous intestinal intussusception. Seventy-three children (11.7%) had in-hospital recurrence of intestinal intussusception after successful reduction with air enema. The baseline characteristics of the study participants are shown in Table 1. There were no significant differences between the in-hospital recurrence group and non-recurrence group in sex, history of previous intussusception, symptom profile, duration of symptoms, site of the intestinal intussusception, mass size, CRP level, WBC count, neutrophil percentage or lymphocyte percentage (Table 1). However, patients with in-hospital recurrence of intussusception were older (median [IQR]: 2.08 [1.67–3.25] vs. 1.67 [0.92–2.67] years-old, *P* < 0.001) and had significantly higher incidences of secondary intussusception (12.3% vs. 0.9%, *P* < 0.001) and mesenteric lymph node enlargement (56.2% vs. 38.1%, *P* = 0.003) than those who did not have recurrence.

**Table 1:** Baseline clinical characteristics of the study participants

Characteristic	All (*n* = 624)	No recurrence (*n* = 551)	Recurrence (*n* = 73)	*P*
Age (years)	1.8 (1.00–2.8)	1.7 (0.9–2.7)	2.1 (1.7–3.3)	< 0.001
≤1 year-old	178 (28.5%)	174 (31.6%)	4 (5.5%)	0.001
>1 year-old	446 (71.5%)	377 (68.4%)	69 (94.5%)	
Male	400 (64.1%)	346 (62.8%)	54 (74.0%)	0.061
Symptoms				
Fever	152 (24.4%)	134 (24.3%)	18 (24.7%)	0.950
Vomiting	333 (53.4%)	295 (53.5%)	38 (52.1%)	0.810
Bloody stools	120 (19.2%)	112 (20.3%)	8 (10.9%)	0.056
History of previous intussusception	74 (11.9%)	61 (11.1%)	13 (17.8%)	0.094
Type of intussusception				< 0.001
Primary	610 (97.8%)	546 (99.1%)	64 (87.7%)	
Secondary	14 (2.2%)	5 (0.9%)	9 (12.3%)	
Site of intussusception				0.806
Hepatic flexure	237 (38.0%)	206 (37.4%)	31 (42.5%)	
Transverse colon	184 (29.5%)	162 (29.4%)	22 (30.1%)	
Ileocecal junction	92 (14.7%)	84 (15.2%)	8 (11.0%)	
Ascending colon	63 (10.1%)	55 (10.0%)	8 (11.0%)	
Splenic flexure	31 (5.0%)	30 (5.4%)	1 (1.4%)	
Other	17 (2.7%)	14 (2.5%)	3 (4.1%)	
Duration of symptoms				0.901
≤2 days	575 (92.1%)	508 (92.2%)	67 (91.8%)	
>2 days	49 (7.9%)	43 (7.8%)	6 (8.2%)	
Mesenteric lymph node enlargement	251 (40.2%)	210 (38.1%)	41 (56.2%)	0.003
Size of mass (cm)				
Inner diameter	2.8 (2.6–3.1)	2.8 (2.6–3.1)	2.9 (2.5–3.2)	0.220
Outer diameter	3.5 (3.1–3.8)	3.5 ± 0.7	3.6 ± 0.8	0.290
CRP (mg/L)	5.1 (1.8–11.4)	5.2 (1.8–11.0)	4.3 (1.8–13.0)	0.907
WBC count (×10^9^/L)	11.0 (8.6–13.6)	10.9 (8.6–13.4)	11.1 (8.6–14.4)	0.655
Neutrophils (%)	62.4 (48.7–73.6)	61.3 ± 15.9	62.3 (46.6–70.9)	0.386
Lymphocytes (%)	30.1 (19.9–41.5)	30.8 ± 13.9	31.6 ± 13.2	0.544

Data are presented as median (IQR), mean ± SD or *n* (%)

### Logistic regression analysis of factors associated with in-hospital recurrence of intussusception

The multivariate analysis identified age > 1 year-old (OR = 7.65, 95%CI = 2.70–21.71, *P* < 0.001), secondary intestinal intussusception (OR = 14.40, 95%CI = 4.31–48.14, *P* < 0.001) and mesenteric lymph node enlargement (OR = 1.90, 95%CI = 1.13–3.18, *P* = 0.015) as factors independently associated with in-hospital recurrence of intussusception (Table 2).

**Table 2:** Multivariate logistic regression analysis of factors associated with in-hospital recurrence of intestinal intussusception

Variable	Odds ratio (95% confidence interval)	*P*
Age (> 1 years-old vs. ≤1 years-old)	7.65 (2.70–21.71)	< 0.001
Type of intussusception (secondary vs. primary)	14.40 (4.31–48.14)	< 0.001
Mesenteric lymph node enlargement (yes vs. no)	1.90 (1.13–3.18)	0.015

## Discussion

The main findings of this retrospective analysis were that age > 1 year-old, secondary intestinal intussusception and mesenteric lymph node enlargement were independently associated with increased odds of in-hospital recurrence of intussusception. We suggest that children with intestinal intussusception who have any of these clinical characteristics should be carefully monitored throughout their hospital stay to ensure that any recurrence of intestinal intussusception after treatment is diagnosed and managed in a timely manner.

In-hospital recurrence of intestinal intussusception occurred in 11.7% of the children included in this study. The incidence in our cohort agrees well with the overall rate of 8–19% described previously [9–13], given that over half of all recurrences occur within 48 h after treatment [14, 17, 22, 23]. The incidence of 11.7% is also broadly consistent with values reported by studies of early recurrence, including those by Lee et al. (in-hospital recurrence of 6.1%) [24], Zhu et al. (9.0% within 72 h of reduction) [28], Shen et al. (12.8% within 72 h of reduction) [27] and Adhikari et al. (16.7% within 1 month of treatment) [30].

The present study found that age > 1 year-old was an independent factor strongly associated with in-hospital recurrence of intussusception (OR = 7.65). This result agrees well with several published studies. For example, Guo et al. and Chen et al. identified age > 1 year-old as an independent predictor of the recurrence of intestinal intussusception [13, 14]. Similarly, Kim et al. and Xie et al. reported that age ≥ 2 years-old was independently associated with recurrent intussusception [15, 16]. Although the above four studies did not focus on in-hospital or early recurrence, other investigations specifically evaluating the early recurrence of intussusception after treatment have also identified older age as a risk factor. Simanovsky et al. concluded that children with recurrence of intussusception within 48 h of treatment were older than those who did not have early recurrence (23 months-old vs. 12 months-old) [25], while Shen et al. concluded that age ≥ 2 years-old was related to short-term recurrence [26]. However, Cho et al. did not find any differences in age between patients with recurrence of intussusception within 48 h and those without recurrence [29], and a similar finding was reported by Adhikari et al., who evaluated short-term recurrence within 1 month of treatment [30]. The reasons for the disparity between studies remain unclear, although it is possible that differences in analytical approach (e.g., grouping on the basis of < 1 year-old vs. <2 years-old) may have contributed. In addition, given that the incidence of recurrence is quite low, it cannot be excluded that some studies were underpowered to detect a real association between age and recurrence of intestinal intussusception.

Secondary intestinal intussusception (i.e., intussusception due to the presence of identifiable organic disease) was strongly associated with disease recurrence in the present study (OR = 14.40). This finding concurs with several prior investigations demonstrating that PLPs are independent predictors of recurrent intussusception [13, 14, 16, 21, 27]. PLPs are associated with 0.3–20% of cases of intestinal intussusception in children [31, 32], with the main causative PLPs being Meckel’s diverticulum, ileal polyp, duplication of the ileum, Peutz–Jeghers syndrome, malignant tumors and appendiceal stump [32]. It is becoming increasingly accepted that PLPs elevate the risk for intussusception recurrence [8], and the present study provides further evidence to support this.

Our analysis also identified mesenteric lymph node enlargement as a predictor of in-hospital recurrence of intestinal intussusception (OR = 1.90). This result agrees with the study of Zhang et al., who found that enlarged abdominal lymph nodes were related to recurrence of intussusception [20]. Adhikari et al. also observed that the incidence of lymph node enlargement was numerically higher in the recurrence group than in the non-recurrence group (61.5% vs. 47.7%), although the difference was not statistically significant [30]. Enlarged lymph nodes have been reported to not only decrease the hydrostatic reduction rate but also act as a lead point in intussusception [33]. Thus, it is possible that the presence of mesenteric lymph node enlargement might act as a PLP to increase the risk of the intussusception reforming after initially successful treatment.

Among the other factors evaluated in this study, an absence of bloody stools (*P* = 0.056) and male sex (*P* = 0.061) were borderline significantly more common in the recurrence group than in the non-recurrence group. With regard to the former, Lee et al. also reported that bloody stools were less common in the recurrence group than in the non-recurrence group [24], whereas Xie et al. conversely found that rectal bleeding was a risk factor for recurrence [16]. With regard to the latter, most studies have not detected an influence of sex on intussusception recurrence, although one analysis suggested a higher risk of recurrence in females [17]. Thus, it seems unlikely that sex and bloody stools are major factors influencing the risk of recurrence of intestinal intussusception.

This study has some limitations. First, this was a retrospective analysis, so the results may have been affected by information bias or selection bias. Second, this was a single-center study, so the generalizability of the findings cannot be confirmed. Third, since the number of patients with recurrence of intussusception was quite small (due to the low risk of recurrence), the study may have been underpowered to detect some real differences between the recurrence and non-recurrence groups. Fourth, other unknown factors not included in the analysis may have influenced the results. Large-scale, prospective, multi-center studies are needed to further evaluate the factors associated with in-hospital recurrence of intestinal intussusception.

## Conclusions

In conclusion, this single-center, retrospective analysis of children treated for intestinal intussusception found that age > 1 year-old, secondary intestinal intussusception and mesenteric lymph node enlargement were independently associated with increased odds of in-hospital recurrence. Children with intestinal intussusception who have any of these clinical characteristics may require close observation during hospitalization to ensure that any recurrence of intussusception is diagnosed and treated in a timely manner.

## Data Availability

All data generated or analyzed during this study are included in this published article.

## Abbreviations

PLPs: Pathological lead points
WBC: white blood cell
CRP: C-reactive protein
SD: standard deviation
ORs: Odds ratios
